# Acyl Homoserine Lactones from Culture Supernatants of *Pseudomonas aeruginosa* Accelerate Host Immunomodulation

**DOI:** 10.1371/journal.pone.0020860

**Published:** 2011-06-16

**Authors:** Ravi Kumar Gupta, Sanjay Chhibber, Kusum Harjai

**Affiliations:** Department of Microbiology, Bio Medical Sciences Block, Panjab University, Chandigarh, India; National Institute of Allergy and Infectious Diseases, National Institutes of Health, United States of America

## Abstract

The virulence of *Pseudomonas aeruginosa* is multifactorial and under the control of quorum sensing signals, such as acyl homoserine lactones (AHLs). The importance of these molecules in the establishment of infection has been previously reported. These molecules either improve the virulence potential of *P. aeruginosa* or modulate the host immune response. To establish the immune modulating potential of quorum sensing signal molecules, previous studies have only used synthetic AHLs. However, there can be differences in the biological properties of synthetic and natural AHLs. The use of naturally extracted AHLs from the culture supernatant of *P. aeruginosa* is likely to simulate natural conditions more than the use of synthetic AHLs. Therefore, in the present study, the immune modulating potential of synthetic and naturally extracted AHLs was compared using a thymidine uptake assay, immunophenotyping and sandwich ELISA in order to assess mouse T-cell proliferation and production of Th1 and Th2 cytokines. Natural AHLs were able to suppress T-cell proliferation, even at low concentrations, compared to synthetic AHLs. The majority of cells undergoing proliferation were CD4+, as revealed by immunophenotyping. The inhibition of T-cells was stronger with natural AHLs compared to synthetic AHLs. Moreover, the natural AHLs were also able to shift immune responses away from host protective Th1 responses to pathogen protective Th2 responses.

## Introduction

Host immune responses are important mechanisms that significantly contribute to the infection process. Different pathogens use different strategies to either escape or modulate host immune responses. The level and nature of immune responses mainly depends upon the type of infection and the site of involvement. Hence, modulation of immune responses through host or pathogen traits can have a bearing on the outcome of an infection. This phenomenon is especially important in immunocompromised hosts, for example, in nosocomial settings. *P. aeruginosa* is a notorious opportunistic pathogen that is difficult to eradicate with conventional antibiotic therapy. The pathogen produces *N*- (3-oxododecanoyl) homoserine lactone (OdDHL) and *N*-butyryl-L-homoserine lactone (BHL) as its QS signal molecules [1]–[2], which collectively regulate 4–12% of the total genome [3]. QS signal molecules promote *P. aeruginosa* infections both by regulating virulence arsenals and by varying host immune responses for pathogen survival [4].

Telford et al. [5] carried out the first direct analysis of the effects of OdDHL on myeloid cells involved in the immune response. OdDHL is known to stimulate various host signalling pathways as a mechanism for inhibiting or activating immune cell responses. The immunomodulatory effect that is subsequently generated has led to the hypothesis that QS modulates T-cell responses away from host protective helper T (Th1) host responses. An increasing body of evidence suggests that OdDHL can interact with different eukaryotic cells, such as epithelial cells and fibroblasts and induce Th2- mediated inflammatory responses as well [6].

In all of the earlier studies, synthetic OdDHL have been employed. Although various studies have reported 98–99% homology between synthetic acyl homoserine lactones (AHLs) and natural AHLs [7], the biological response to synthetic AHLs may not be the same to that observed with natural AHLs. To date, studies that have assessed the biological response to synthetic AHLs have relied on bioassays that use specific reporter strains. It is likely that the biological response generated towards synthetic AHLs and natural AHLs produced by wild-type *Pseudomonas* may in-fact be different in natural hosts, where AHLs interact with several different types of cells and environmental conditions. The stereochemistry of synthetic AHLs can also be different from that of natural AHLs. For example, all natural acyl-HSLs are (*S*) enantiomers, while synthetic AHLs are (*R*) enantiomers [8]–[9]. The present study was designed to compare the immunological responses of T-cells exposed to naturally extracted bacterial AHLs and synthetic AHLs *in vitro*. For this purpose, extracted bacterial culture supernatants were used as a source of natural AHLs to evaluate the effect of AHLs on the proliferation of murine splenocytes and the production of a Th1 and Th2 cytokine response. Synthetic AHLs were used as controls.

## Results and Discussion

QS signal molecules promote *P. aeruginosa* infections both by regulating the expression of virulence factors as well as by varying host inflammatory and immune responses [10]. Host immune responses to *P. aeruginosa* infections have been shown to be variable in experimental models [11]. The present study provides evidence showing the immunomodulatory properties of natural QS signal molecules extracted from bacterial culture supernatants. Previous studies have reported the immunomodulatory properties of synthetic AHLs by using high concentrations of synthetic QS signal molecules [5], [12]. In the present study, the effect of natural AHLs extracted from bacterial culture supernatant was compared to that of pure synthetic OdDHL and BHL on T-cell (mouse splenocytes) proliferation and cytokine production. Although the exact concentration of AHLs that is achievable in tissues is unknown, it is likely that it may be in the range of 0.1 µm to 30 µm *in vivo*. Therefore, we employed different concentrations of synthetic AHLs as well as naturally extracted QS signal molecules in the range of 0.1 µm to 30 µm to simulate the possible available concentrations of AHLs *in vivo*.

**Figure 1:**
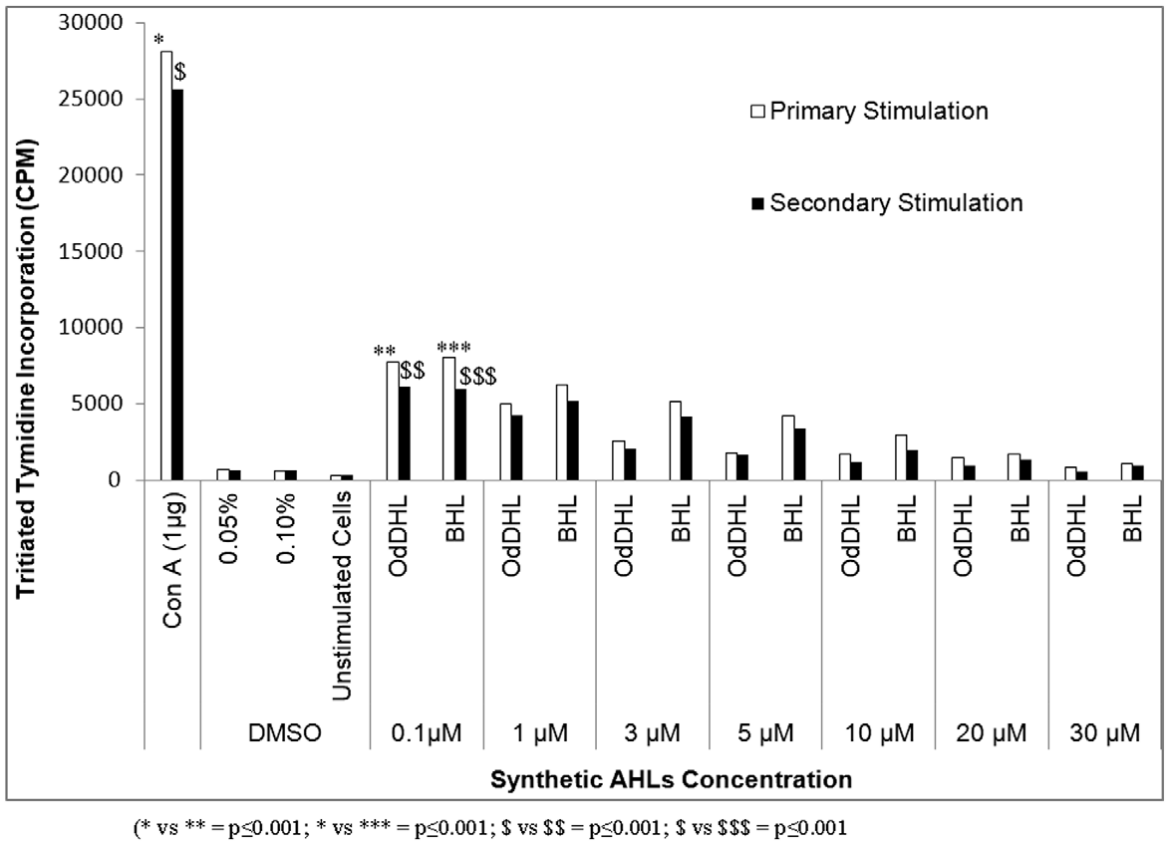
Results showing the uptake of thymidine by actively growing cells in respective experiments of primary and secondary stimulation. Splenocytes were incubated with different concentrations of synthetic AHLs (OdDHL and BHL) at 37°C for 48 h in the presence of 5% CO_2_ and were counted on the basis of expression of incorporated thymidine. For secondary stimulation, cells were exposed to mitogen ConA (1 µg), followed by a subsequent exposure to different concentrations of synthetic AHLs. Unstimulated and DMSO treated cells were used as controls. Results are expressed as mean values ± SD of counts per minute for proliferating cells in the cell culture. The asterisk indicates the level of significance.

**Figure 2:**
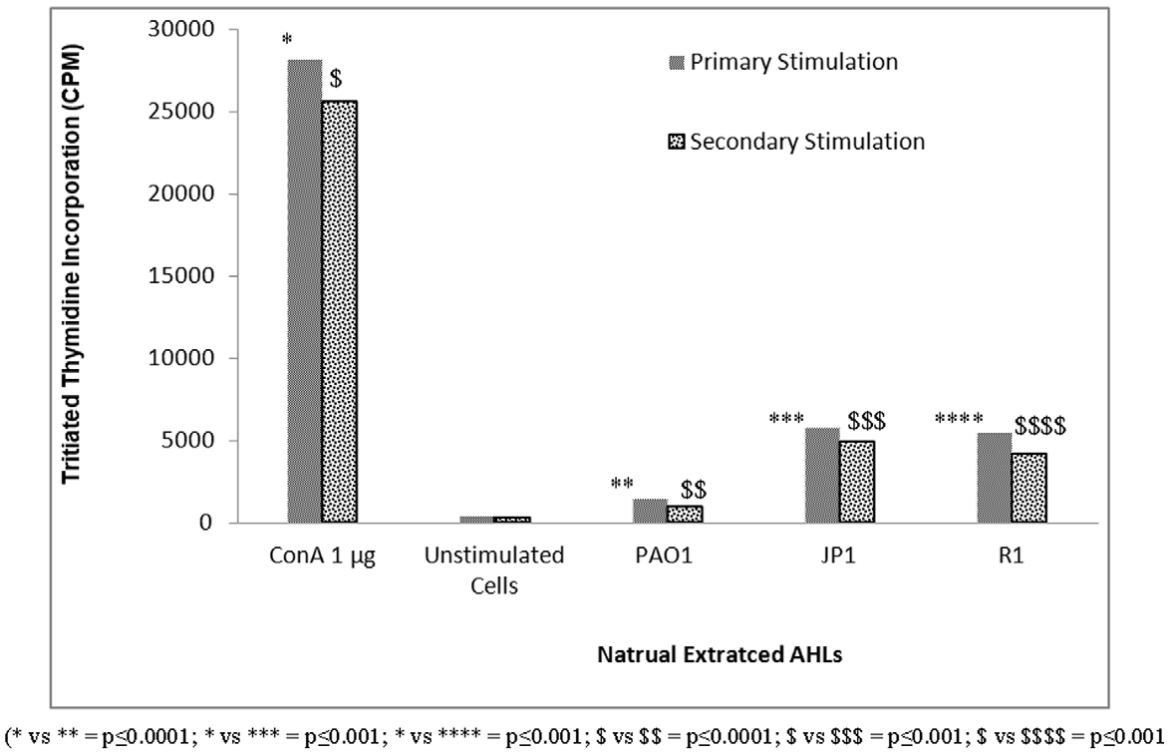
Results showing the uptake of thymidine by actively growing cells in respective experiments of primary and secondary stimulation. Splenocytes were incubated with natural AHLs extracted from the wild-type standard strain PAO1 and its quorum sensing mutant strains JP1 and R1 (2.5 µM) at 37°C for 48 h in the presence of 5% CO_2_. For secondary stimulation, cells were first exposed to mitogen ConA (1 µg), followed by exposure to natural AHLs. Unstimulated cells were used as experimental control. Results are expressed as mean values ± SD of counts per minute for cells under proliferation in cell culture. The asterisk indicates the level of significance.

The mitogen ConA showed maximum T-cell proliferation when used at a concentration of 1 µg, whereas synthetic AHLs, OdDHL and BHL, inhibited T-cell proliferation at a much lower concentration (0.1 µm). In comparison to ConA, significantly fewer T-cells underwent proliferation after primary and secondary stimulation with synthetic AHLs (p≤0.001) (Figure 1). There was a significant decline in the number of proliferating T-cells (p≤0.001) after the concentration of OdDHL and BHL was increased (from 1 µm to 30 µm), suggesting a dose-dependent rate of inhibition. Earlier studies reported a similar dose-dependent inhibition of mouse splenocyte proliferation with high concentrations of OdDHL [5], [7]. Boontham et al. [12] also observed the inhibitory effect of OdDHL (10–100 µm) on dendritic cell proliferation from patients and healthy hosts. However, control cells incubated with DMSO (AHL solvent) in this study did not show any significant T-cell proliferation (p≤0.0001). OdDHL showed a strong inhibitory effect even at low concentrations compared to BHL, which showed comparable results only at a higher concentration (p≤0.001), suggesting that OdDHL was more effective at inhibiting T-cell proliferation than BHL. Complete inhibition of splenocytes by synthetic AHLs occurred at high concentrations (10–30 µm), whereas natural AHLs from wild-type PAO1 completely inhibited cell proliferation at a low concentration of 2.5 µm (p≤0.01) (Figure 2). This might be due to the combined effect of both AHLs, since the wild-type strain produced OdDHL and BHL simultaneously, or could be due to differences in the stereochemistry of both synthetic and natural QS signal molecules. In an earlier study, Pomini et al. [13] reported differences in the stereochemistry of synthetic and natural AHLs. Although naturally extracted AHLs from QS mutant strains JP1 and R1 showed significant inhibition of T-cell proliferation (p≤0.001) compared to the mitogen ConA, this effect was comparatively lower than that obtained with natural AHLs extracted from the wild-type strain. This difference can be attributed to the action of BHL alone, since extracts of these mutants lacked OdDHL. In addition, synthetic OdDHL has been shown to be more effective than BHL in previous studies. These results confirm that AHLs in combination are more effective in inducing T-cell proliferation (as in the case of the wild-type strain) than individual AHLs (BHL in the mutant strains).

Natural and synthetic AHLs acted as secondary stimulants, whereas ConA, a T-cell specific mitogen, was a primary stimulant. Since AHLs can affect T-cells during the first 2 h of culture [14], the addition of AHLs during the 2 h period of primary stimulation with ConA resulted in decreased T-cell proliferation. The presence of both natural and synthetic AHLs caused a significant decrease in the counts of proliferating cells during secondary stimulation (p≤0.001). This is close to the *in vivo* environment during the natural infectious cycle, where priming of immune cells takes place initially with antigens, such as LPS or other virulence factors of the pathogen. The results of the present study suggest that AHLs could still have the ability to modulate the host immune responses by modulating the already activated immune system in such conditions. This ability of AHLs may contribute towards the survival of *P. aeruginosa in vivo* as a successful pathogen. Natural AHL molecules of *P. aeruginosa* may thus help in the pathogenesis of the bacterium by modulating the host immune responses and subsequently leading to chronic and persistent infections.

**Figure 3:**
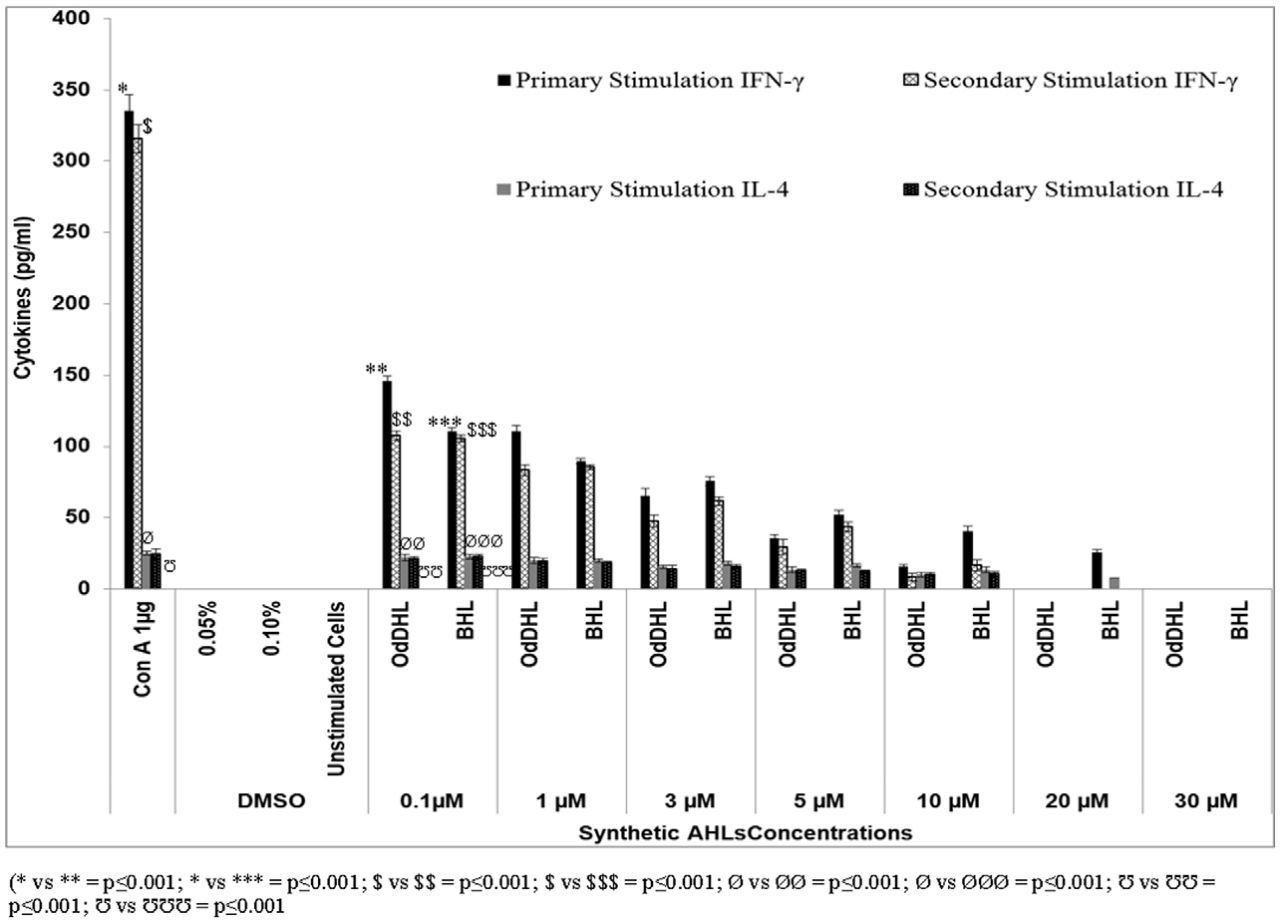
The level of cytokines, IFN-γ and IL-4, as estimated by a sandwich ELISA in T-cell culture supernatants during primary and secondary stimulation with synthetic AHLs (OdDHL and BHL). During secondary stimulation, cells were first exposed to mitogen ConA followed by stimulation with different concentrations of OdDHL and BHLs. Unstimulated and DMSO treated cells were used as control. Results are expressed as mean values ± SD of cytokine levels (in pg/ml) in the cell culture supernatants. The asterisk indicates the level of significance.

**Figure 4:**
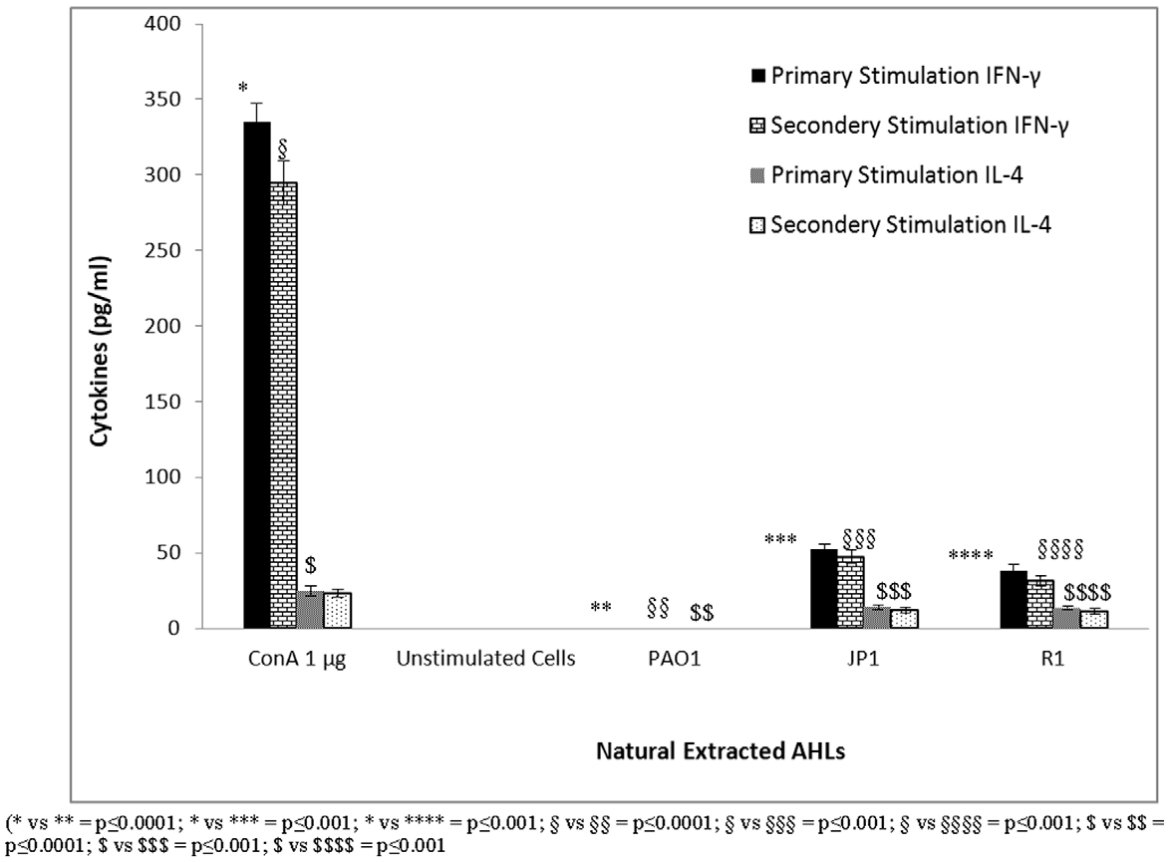
The level of cytokines, IFN-γ and IL-4, as estimated by a sandwich ELISA in T-cell culture supernatants during primary and secondary stimulation with natural AHLs extracted from the wild-type strain PAO1 and its isogenic quorum sensing mutant strains JP1 and R1. During secondary stimulation, cells were first exposed to mitogen ConA followed by stimulation with natural AHLs. Unstimulated cells were used as a control. Results are expressed as mean values ± SD of cytokine levels (in pg/ml) in the cell culture supernatants. The asterisk indicates the level of significance.

In order to gain insight into the involvement of the cell lineage, immunophenotyping of cultured cells stained with anti-CD4+/CD8+ monoclonal antibodies (BD Biosciences, USA) was performed. The results showed that the cells undergoing the most proliferation were CD4+ cells. These CD4+ cells underwent further differentiation and produced Th1 and Th2 type specific T-cells. A sandwich ELISA was performed to estimate the amount of Th1 and Th2 specific cytokines present in the cell culture supernatant following T-cell proliferation. Levels of IFN-γ (Th1 cytokine) and IL-4 (Th2 cytokine) were below the detection limits in supernatants obtained from a normal T-cell population without any antigenic stimulation or upon induction with DMSO *in vitro*. However, when ConA was added, a maximum production of both IFN-γ and IL-4 was achieved. Upon stimulation with 1 µg of ConA, splenocytes produced 335 pg/ml and 315 pg/ml of IFN-γ and 25 pg/ml and 22.5 pg/ml of IL-4 after primary and secondary stimulation, respectively (Figure 3). Induction with a low concentration (0.1 µm) of OdDHL and BHL showed significantly low levels of both Th1 and Th2 cytokines (IFN-γ and IL-4) (p≤0.001). Additional incubation with these two agents showed a dose-dependent reduction in cytokine production. These results corroborate the findings of previous studies in which the exposure of mouse and human immune cells to AHL led to decreased levels of cytokines (IFN-γ and IL-4) [12], [14], [15]. Higher concentrations of both AHLs affected IL-4 production. In comparison, OdDHL suppressed Th1 and Th2 cytokine responses during primary and secondary cell stimulation more effectively than BHL. BHL was an effective inhibitor only when employed at a higher concentration. Natural AHLs extracted from PAO1 induced cytokine levels that were markedly below the limit of detection, indicating that no cell proliferation occurred. When the cells were stimulated with natural extracts of AHLs from wild-type PAO1, the production of IFN-γ and IL-4 was completely inhibited (p≤0.0001, Figure 4). Similarly, stimulation with extracts from the QS mutant strains significantly inhibited cytokine production in culture supernatants from the T-cell culture, compared to stimulation with mitogen ConA (p≤0.001). The QS mutant strains did not show complete inhibition as observed with the wild-type strain. Naturally extracted AHLs from wild-type and mutant strains also showed similar results during secondary stimulation. However, the mechanism of cell growth and cytokine inhibition needs to be explored further.

The results of the present study have revealed that AHLs affected IFN-γ (Th1 cytokine) production more than IL-4 (Th2 cytokine) production. This suggests that AHLs can modulate Th1 immune responses and therefore help the pathogen to establish in the host even at a concentration as low as 0.1 µm. Suppression of IL-4 at higher concentrations of OdDHL and BHL also suggests that they have less effect on Th2 type cells. On the other hand, Th1 type cells showed more susceptibility to synthetic as well as natural AHLs compared to Th2 type cells. These findings suggest that the presence of AHLs can significantly modulate the immune response by redirecting the response from a Th1 to Th2 type. Th1 dominated immune responses are known to have better immune function compared to Th2 dominated immune responses [15]–[16]. In the case of secondary stimulation with synthetic and natural AHLs, a similar trend in the production of both the cytokines was observed, as noted during primary stimulation. However, enhanced suppression of cytokines during secondary stimulation further suggests that the production of IFN-γ, which is a host-protective cytokine, is down-regulated. The earlier reports also show a shift from Th1 to Th2 response following exposure to synthetic OdDHL [5], [17]. The ability of AHLs to support a Th2 immune response suggests that this is a significant bacterial defense strategy for combating infections. Th2 responses dysregulated the antibacterial Th1 immune responses, which in turn support cell-mediated immunity. A Th1 type of immune response is especially important in defending the host against invading bacteria in the early stage of an infection. The suppression of Th1 responses with naturally extracted AHLs in this study suggests the survival of *P. aeruginosa in vivo*. This might be due to the dysregulation and dysfunction of host defence mechanims, which can subsequently lead to chronic *P. aeruginosa* infections.

The effective immunomodulation by AHLs in the present study suggests that these molecules can divert the host immune response away from a host protective to pathogen protective response. Natural AHLs were found to be more effective than synthetic AHLs at comparable concentrations *in vitro*. These results reveal that the relevant and absolute configuration of acyl-HSLs is important for their ideal biological response in Gram-negative bacteria. However, to the best of our knowledge, assessment of the absolute configuration of acyl-HSLs from natural AHLs has not been attempted as yet [18], [9]. In addition, the screening of synthetic AHLs for biological responses *in vitro* through biosensors does not confirm that their *in vivo* responses will be similar [13], since biosensors can neither distinguish nor confirm the conformational changes between synthetic and natural AHLs. Although some authors have used synthetic OdDHL for *in vivo* and *in vitro* experiments, such experiments cannot rule out the toxicity of synthetic compounds and responses *in vivo* due to conformational changes [5]–[7], [12], [16]. The results of the present study show that one should preferably use naturally extracted AHLs for *in vitro* studies for the true evaluation of biological properties of AHLs *in vivo*. Moreover, the effect of synthetic OdDHL on immune cells, as reported in literature, may not simulate a similar effect *in vivo*. Therefore, the utmost caution should be taken when extrapolating the results of *in vitro* studies using synthetic AHLs to the expected *in vivo* effects. In conclusion, slight differences in the stereochemistry [19] of both synthetic and naturally extracted AHLs can create different responses. These results suggests that naturally extracted AHLs from bacterial culture supernatants are more active and should be used for *in vitro* experiments at concentrations that are achievable *in vivo*.

## Materials and Methods

### Bacterial Strains

The standard *P. aeruginosa* strain PA01 and its isogenic mutant strains JP1 (Δ*LasI*) and R1 (Δ*LasR*) were used in this study. Tetracycline (50 µg/ml) was used for the maintenance of JP1 and R1 mutant strains. These mutant strains were created by gene replacement and were generously provided by Prof. Barbara H. Iglewski, University of Rochester, New York, USA.

### Extraction of AHLs from culture supernatant

Briefly, bacterial cells were removed by centrifugation from cultures grown overnight. Culture supernatants were extracted twice with an equal volume of acidified ethyl acetate [20]. The combined extracts were dried over anhydrous magnesium sulphate and the residues were resuspended in 50–100 µl of HPLC grade ethyl acetate. Extracted AHLs were made endotoxin free (Detoxi-gel endotoxin removing gel, Thermo Scientific, USA) and were subjected to an LAL test to confirm the absence of bacterial endotoxin. Synthetic standard OdDHL, BHL (0.1 µm to 30 µm), and ethyl acetate extracted culture supernatants (natural extracted AHLs, 2.5 µm) were used for the activation of T-cells *in vitro*. The concentration of AHLs was estimated using standard synthetic OdDHL and BHL as a reference.

### Spleen cell cultures

Spleens were removed from LACA female mice. The ethical clearance was obtained from the Institutional Ethics Committee for using experimental animals. Single cell suspensions (splenocytes) were prepared and erythrocytes were removed by incubation in red cell lysis solution (0.9% NH_4_Cl, Sigma Chemicals, USA) for 1 min on ice, followed by centrifugation [16]. The pellet was then resuspended in cell culture media (CCM). All splenocyte cultures were incubated at 37°C in 5% CO_2_ at a concentration of 1×10^6^cells/ml. The cells were incubated in 2 -ml volumes of CCM in 24 -well tissue culture plates (Greiner GmbH, Germany).

### Primary and secondary stimulation of splenocytes

For primary stimulation, splenocytes from mice were stimulated with OdDHL and BHL at concentrations ranging from 0.1 to 30 µm. The natural AHLs extracted from the bacterial culture were used at a concentration of 2.5 µm as indicated for the individual experiment. The spleen cells stimulated with mitogen ConA (Sigma Chemicals, USA) at 1, 3 and 5 µg were used as a control. All of the cultures were incubated in 5% CO_2_ at 37°C for 48 to 72 h. For primary and secondary stimulation, a proliferation assay was performed in the presence of ConA, AHLs, or DMSO (0.05% and 0.1%) for the indicated time periods shown for each experiment. For secondary stimulation, cells exposed earlier to mitogen were incubated with different concentrations of AHLs together with their respective controls. Supernatants from all of the cultures were harvested for the measurement of cytokine production by sandwich ELISA, whereas the cells were used for the thymidine uptake assay. For the uptake assay, cells were incubated with tritiated thymidine and counted in a Perkin Elmer Liquid Scintillation System. The results were expressed as counts per minute (CPM) of active and proliferating cells with incorporated thymidine in the DNA.

### Estimation of T-cell cytokines

Levels of IFN-γ (Th1 cytokine) and IL-4 (Th2 cytokine) in cell culture supernatants were assessed by using ELISA kits (BD BioSciences, USA) according to the manufacturer's instructions. Ninety-six-well microtiter plates (Falcon Corp., USA) were coated with 100 µl of a suitable capture antibody per well. The plates were then coated with 100 µl of cell culture supernatants and incubated at room temperature for two hours. The plates were then washed with wash buffer and incubated with streptavidin antibodies followed by incubation with biotinylated antibodies. Plates were incubated in the dark with TMB substrate after washing. Once adequate color developed, the reaction was stopped by adding stop buffer. ELISA plates were read at 450 nm using Microplate Manager 5.1 (BioRad Labs Ltd. USA). Cytokine levels were estimated by using the standard recombinant cytokine supplied with the kits as a reference.

### Ethical Statement

This study was carried out in strict accordance with the recommendations in the Guide for the Care and Use of Laboratory Animals of the National Science Academy through Indian Council of Medical Research, Govt. of India. The protocol was approved by the Committee on the Ethics of Animal Experiments of the Panjab University, Chandigarh (Registration no. CPCSEA, 45/1999 dated 10/2/2000). All the surgical interventions were performed under sodium pentobarbital anaesthesia, and all efforts were made to minimize suffering.

### Statistical Analysis

All the experiments were carried out in triplicate and results were analysed statistically by employing Student's *t* test using SPSS 11.05.
